# Dynamic, heterogeneous endothelial Tie2 expression and capillary blood flow during microvascular remodeling

**DOI:** 10.1038/s41598-017-08982-z

**Published:** 2017-08-22

**Authors:** Molly R. Kelly-Goss, Bo Ning, Anthony C. Bruce, Daniel N. Tavakol, David Yi, Song Hu, Paul A. Yates, Shayn M. Peirce

**Affiliations:** 1 Department of Biomedical Engineering, University of Virginia, Charlottesville, VA USA; 2 Department of Ophthalmology, University of Virginia, Charlottesville, VA USA

## Abstract

Microvascular endothelial cell heterogeneity and its relationship to hemodynamics remains poorly understood due to a lack of sufficient methods to examine these parameters *in vivo* at high resolution throughout an angiogenic network. The availability of surrogate markers for functional vascular proteins, such as green fluorescent protein, enables expression in individual cells to be followed over time using confocal microscopy, while photoacoustic microscopy enables dynamic measurement of blood flow across the network with capillary-level resolution. We combined these two non-invasive imaging modalities in order to spatially and temporally analyze biochemical and biomechanical drivers of angiogenesis in murine corneal neovessels. By stimulating corneal angiogenesis with an alkali burn in Tie2-GFP fluorescent-reporter mice, we evaluated how onset of blood flow and surgically-altered blood flow affects Tie2-GFP expression. Our study establishes a novel platform for analyzing heterogeneous blood flow and fluorescent reporter protein expression across a dynamic microvascular network in an adult mammal.

## Introduction

Angiogenesis involves network level coordination of individual cellular behaviors accomplished through alterations in biochemical and biomechanical signals in the local environment^1–3^. These signaling mechanisms are driven by the initial onset of, and subsequent changes in, blood flow and oxygenation throughout the angiogenic vascular network^4^. At any point in time, the combination of biomechanical and biochemical signals varies across the vasculature, and may potentially give rise to endothelial heterogeneities^5^. While these combined inputs have been evaluated in both cultured endothelial cells and in large vessels, they have not yet been studied in the context of cell heterogeneity in small blood vessels. We developed a novel *in vivo* platform to quantify changes in blood flow across individual capillaries throughout a microvascular network and in the same microvessels where changes in endothelial gene expression were also recorded non-invasively over time. Our platform makes use of photoacoustic microscopy (PAM) and intravital confocal microscopy (ICM) to image the well-established murine cornea angiogenesis assay^6^, wherein networks of neovessels can be dynamically observed.

Using this platform, we found that endothelial Tie2 expression is heterogeneous across the newly-established microvascular network of the cornea. Tie2 is a receptor tyrosine kinase whose phosphorylation is induced by Angiopoietin-1(Ang-1), which activates the Ras/MAP kinase pathway and has been shown to affect endothelial cell adhesion, differentiation, and/or survival, as well as pericyte-endothelial interactions and neovessel maturation^7–12^. There is a substantial body of literature that describes hemodynamic control over endothelial cell protein expression in large vessel endothleium^13–19^, and compelling data from *in vitro* studies on cultured endothelial cells has suggested that shear stress is a regulator of Tie2 signaling and vascular quiescence^20, 21^. However, this relationship has never before been corroborated by *in vivo* studies, despite the ongoing clinical evaluation of a drug, VE-PTP, intended to constitutively block this pathway’s inhibitor. Additionally, multiple groups have leveraged complicated computational modeling techniques to investigate endothelial heterogeneity, without the detailed spatial and temporal data on Tie2 gene modulation in tandem with hemodynamic readouts^18, 22–26^. Given that blood flow is dynamic and highly variable from vessel to vessel^27^, we hypothesized that heterogeneity in endothelial cell Tie2-driven green fluorescent protein (GFP) expression during growth and remodeling of the microvascular network may, in part, result from changes in blood flow-induced wall shear stress (WSS) during angiogenesis^28–30^.

To test this hypothesis, we employed ICM to visualize GFP expression in adult transgenic Tie2-GFP fluorescent reporter mice and PAM to quantify blood flow and WSS in the angiogenic network over time, as well as before and after surgically redistributing blood flow in the network. Our experimental platform enables the first *in vivo* investigation into correlated heterogeneities in endothelial protein expression and microvessel hemodynamics throughout a microvascular network. As we executed our study in the murine cornea, Table 1 conveys the advantages of our platform over other experimental approaches and demonstrates how broadly generalizable our method is to other model systems of angiogenesis, such as tumor vascularization and hydrogel scaffold compatibility assays^6, 31–39^. Further, our platform has broader applicability to elucidate the pathogenesis of microvascular disease, such as that associated with diabetes and obesity, where the interplay between hemodynamics and gene expression heterogeneities during pathologic vascular processes may also underlie the success or failure of potential therapeutic treatments.

**Table 1:** Models of *In Vivo* Corneal Angiogenesis.

Experimental Model	Investigation focus	Intravital imaging modality*	Vascular network architecture	Single-cell resolution	Protein expression	Serial imaging	Blood velocity	sO_2_	Distinguish depth of vascular bed	Citation
Micropocket (tumor)	Tumor vascularization	Bright field microscopy	✓			✓				36, 37
Micropocket (growth factor)	Evaluation of angiogenic growth factors in multiple mouse genotypes	Slit lamp microscopy	✓			✓				38
Micropocket (pancreatic islet)	Pancreatic islet vascularization; β-cell function and rate of death	Confocal microscopy	✓	✓	✓					39
Micropocket (adipose-derived stem cells (ASCs))	ASCs in HLA scaffolds; corneal regeneration	Bright field microscopy			✓					40
Micropocket (hydrogel scaffold)	Live imaging of biomaterials (compatibility, vascularization)	Confocal microscopy	✓	✓		✓	✓			41
Topical treatment (anti-VEGF)	Anti-angiogenic therapy (human trial)	Bright field microscopy	✓			✓				42
Suture	Lymph-angiogenesis	Confocal microscopy	✓	✓	✓	✓				11
Chemical burn	Detect vessels in cornea with PAM	Photoacoustic microscopy	✓						✓	43
Chemical burn	EC Tie2 expression	Photoacoustic and confocal microscopy	✓	✓	✓	✓	✓	✓	✓	Present study

*Used at the highest resolution.

## Results

### Imaging of capillary hemodynamics and endothelial cell protein expression

**Figure 1:**
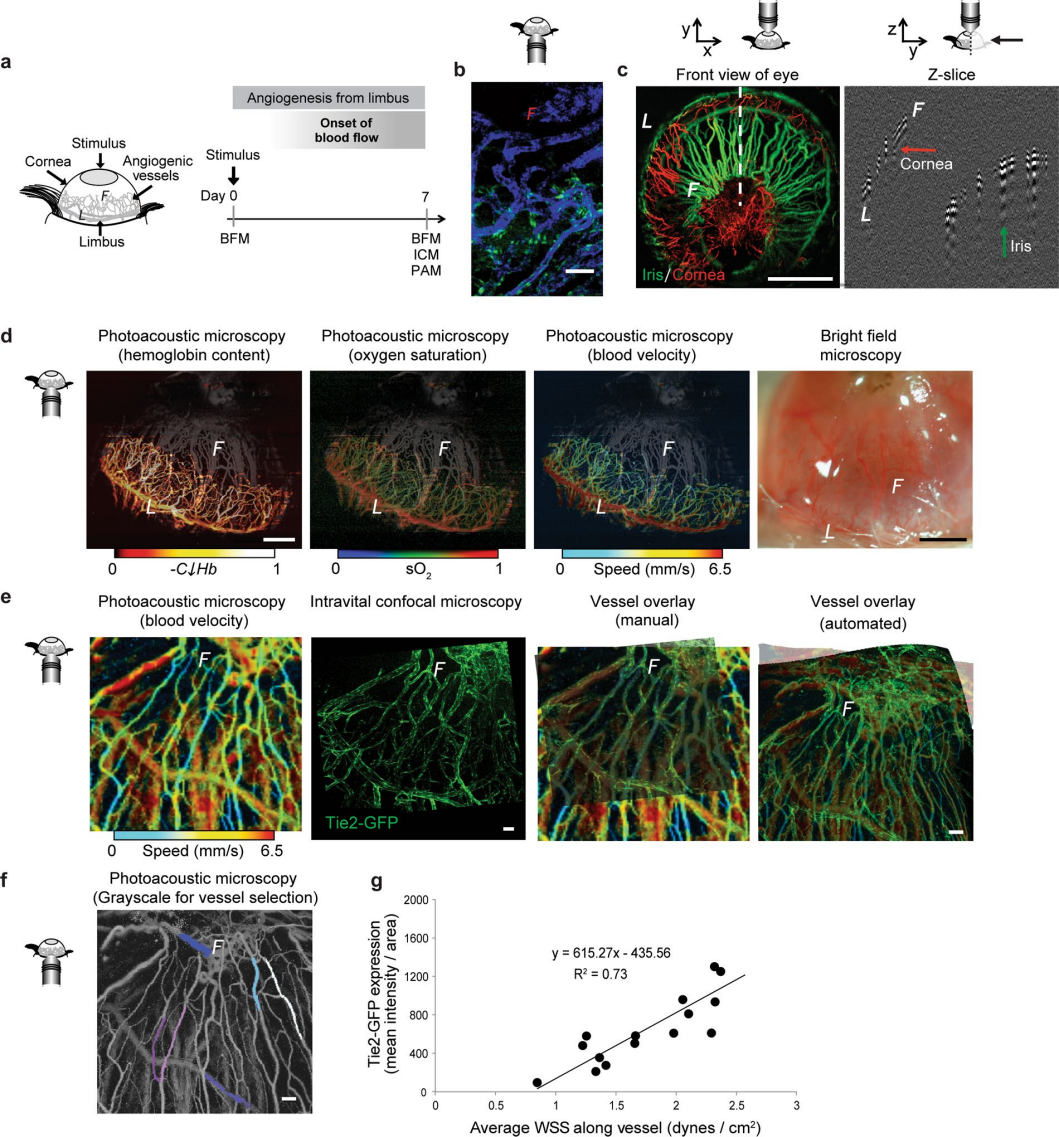
Functional imaging of vascular and cellular activity. (**a**) Schematic of corneal angiogenesis assay in which blood vessels grow from the limbus (“*L*”) into the avascular cornea, creating an angiogenic front (“*F*”) as they develop toward an angiogenic stimulus (gray oval). (**b**) Fluorescence intensity of Tie2-GFP (green) along vessels (perfused with IB4-lectin-647, blue) throughout the network (day 7 shown). (**c**) PAM distinguishes between the iris (green) and corneal (red) vasculatures, due to their different depths. Green arrow points toward iris vessels; red arrow toward corneal neovessels in the Z-slice. (**d**) Unlike BFM, PAM permits functional imaging of relative hemoglobin content (“C_Hb_”), sO_2_ saturation, and blood velocity in the angiogenic corneal network (day 7 shown). The iris vasculature, pseudo-colored in gray based on vessel depth, is visible in the background of each image. (**e**) Overlayed images of the same field of view acquired at day 7 using PAM (blood flow velocity) and ICM (GFP fluorescence intensity), done both manually and with automated software (PT Gui) enables correlation between blood flow-induced wall shear stress (WSS) and GFP fluorescence intensity. (**f**) Grayscale image of same PAM field of view as shown in “d” to facilitate vessel identification. Pseudo-coloring of vessels (white, dark blue, light blue, pink, purple, fuchsia) to exemplify those that were quantified for evaluation in the study. (**g**) Tie2-GFP fluorescence intensity in individual vessels versus average WSS in those vessels calculated using blood velocity maps obtained with PAM, such as those shown in “d”. R^2^ = 0.73 for comparison of 18 vessel segments from four mice. Small schematics next to each microscopy image indicate the direction of the objective relative to the cornea during imaging. Scale bars indicate (**c**,**d**) 1 mm, (**b**,**e**,**f**) 50 µm.

By combining PAM and ICM, we can obtain concurrent information about hemodynamics in capillary segments and protein expression at the cellular level throughout an *in vivo*, mammalian angiogenic network. To demonstrate this, we applied a silver nitrate alkali burn to the central cornea to stimulate angiogenesis from the limbal vessels of 8-week-old Tie2-GFP mice (Fig. 1a). Angiogenesis occurred over seven days, and the new vascular network was imaged using multiple imaging modalities. Standard bright field microscopy (BFM) enabled macroscopic analysis of the developing vascular network. ICM enabled us to visualize GFP expression in endothelial cells (Fig. 1b). PAM provided structural details of the developing vasculature with higher spatial resolution than BFM, as well as functional details of blood flow including relative hemoglobin concentration (C_Hb_), oxygen saturation (sO_2_), and blood velocity.

Further, PAM enables visualization of individual capillaries throughout both the corneal and iris vasculatures due to its ability to image with high-resolution in the x, y, and z directions (Fig. 1c). By analyzing individual cross-sectional images (Fig. 1c, right panel), we separated the iris (green) and corneal (red) vessels to distinguish between the two vascular beds (Fig. 1c, left panel). Using an image processing algorithm developed in MATLAB, we pseudo-colored iris vessels in grey and corneal vessels using a colormetric scale to indicate hemodynamic parameters (Fig. 1d). Specifically, hemoglobin content, oxygen saturation, and blood velocity in individual vessel segments throughout the entire neovascular network were plotted using a colormetric scale (Fig. 1d). PAM provided numerous advantages over BFM (Fig. 1d, right panel), including improved contrast, removal of surface reflections, and the ability to delineate corneal neovessels from iris vessels.

Using ICM, we imaged Tie2-GFP fluorescence intensity in the same corneal neovessel segments that were also imaged using PAM (Fig. 1e, first and second panels from left). The ICM image was manually overlayed onto the blood flow velocity map provided by PAM by visually aligning vessel segments (Fig. 1e, third panel from the left). Additionally, automated projection of PAM onto ICM images using PT Gui software (see Methods) was achieved by generating overlapping alignment pair control points in the two images at locations where vessels branched, in a manner that permitted adjustment of the orientation (roll, pitch, and yaw) and the field of view in the two imaging modalities (Fig. 1e, right-most panel). Individual neovessel segments were manually selected in the PAM image (Fig. 1f), and the fluorescence intensity of Tie2-GFP (indicative of endothelial cell Tie2 expression levels^40^) within those neovessel segments was then quantified. Blood flow velocity in the selected neovessel segments, as measured with PAM, was converted to blood flow-induced WSS according to Eq. 1 (Methods). Plotting Tie2-GFP fluorescence intensity versus blood flow-induced WSS for individual corneal neo-vessel segments revealed a direct linear correlation between Tie2-GFP and WSS (Fig. 1g, R^2^ = 0.73, n = 15 individual neovessel segments from 4 mice). In order to consider the variance across segments, we calculated the ratio of Tie2-GFP expression to WSS at each data point to determine that the overall variance in this ratio was 8.5% across the network (Tie2-GFP/WSS = 336.9 ± 28.78 a.u., mean ± standard error).

### Imaging of capillary hemodynamics and endothelial cell protein expression after surgical redistribution of blood flow

**Figure 2:**
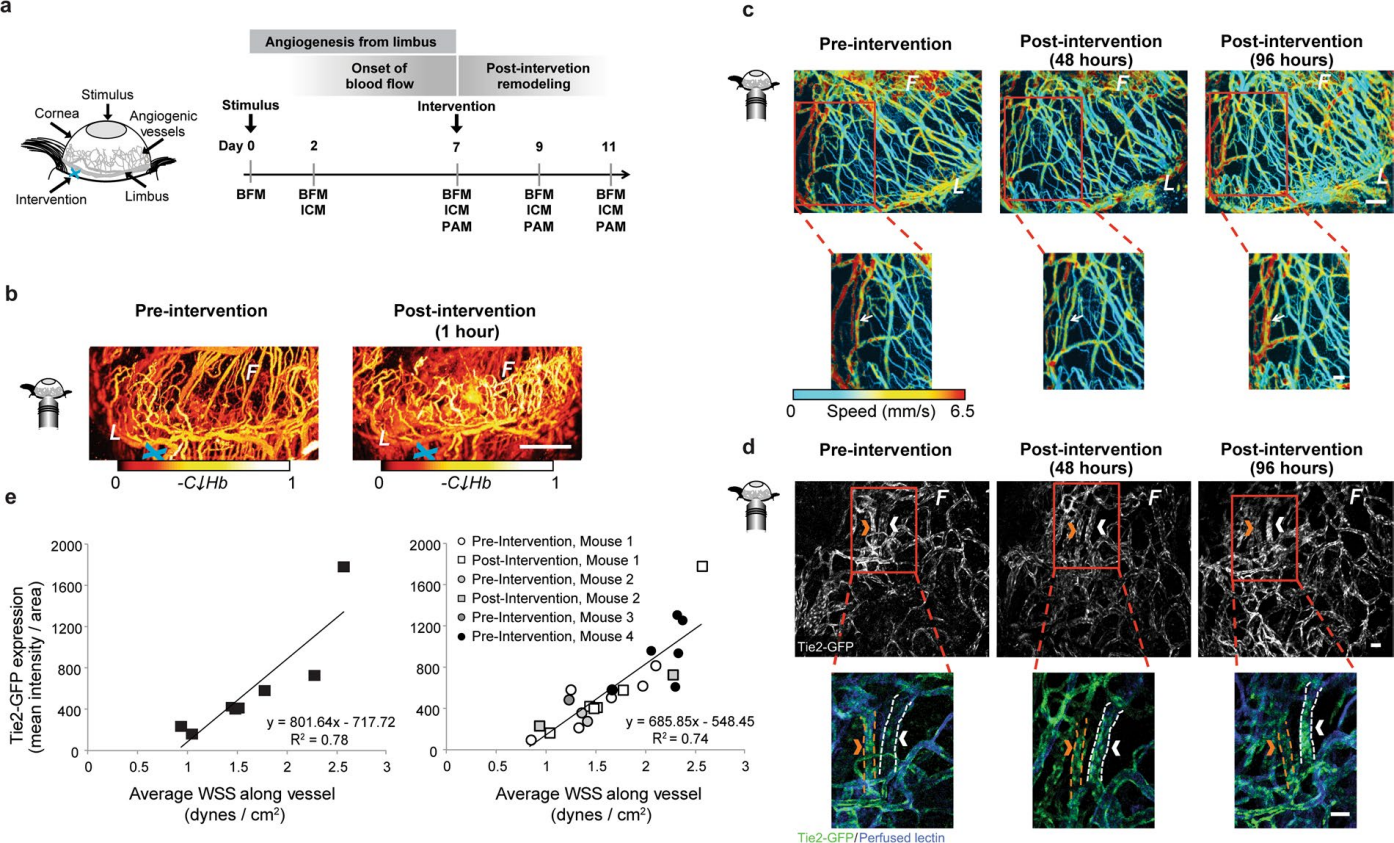
Surgical intervention caused a redistribution in blood flow that resulted in a quantifiable change in blood velocity, flow distribution, and Tie2-GFP fluorescence intensity over time throughout the network. (**a**) Schematic represents the corneal angiogenesis assay in which blood vessels grow from the limbus (“*L*”) into the avascular cornea, creating an angiogenic front (“*F*”). Surgical “intervention” (blue “x”) was performed by applying thermal cautery to an arteriole that feeds into from the limbus. (**b**) PAM images showing relative hemoglobin content (“C_Hb_”) of corneal vessels pre-intervention and one hour post-intervention. (**c**) Dynamic changes in blood flow were visible using PAM in single capillary segments. Bottom row shows a magnified view of the regions boxed in red above. Changes in blood flow velocity (mm/s) are evident in individual vessel segments; white arrow indicates to one segment where flow speed was initially reduced and then returned to pre-intervention levels by 96 hours post-intervention. (**d**) Tie2-GFP fluorescence captured with ICM in single capillary segments over time. Bottom row shows a magnified view of the regions boxed in red above. Changes in Tie2-GFP fluorescence intensity within individual vessel segments are evident between time points. Arrows indicate the same vessel at each time point. Orange arrow indicates a vessel with decreased GFP fluorescence intensity at 96 hours post-intervention; white arrow indicates vessel with increased GFP fluorescence intensity 96 hours post intervention. In the bottom row, Tie2-GFP is green, and perfused IB4-isolectin is blue. (**e**) Vessels with higher WSS exhibited increased Tie2-GFP fluorescence intensity post-intervention. Data in graph on left are from vessels from two mice post-ligation (slope and R^2^ indicated); data in the graph on the right are from vessels assessed both pre- and post-ligation (slope and R^2^ indicated) from multiple mice. White scale bar indicates (**b**) 1 mm, (**c**,**d**) 50 µm.

Previous studies of corneal angiogenesis, using a variety of techniques to induce neovessel growth, have measured either protein expression or capillary hemodynamics (Table 1). However, no study to date has measured changes in capillary hemodynamics alongside changes in protein expression at the level of a single vessel segment or over time. To fill this void, we modified an established vessel ligation technique^37, 41^ to alter blood flow in limbal vessels, and we applied this technique to the corneal neovasculature to demonstrate the ability to measure changes in capillary hemodynamics and endothelial cell protein expression in individual neovessel segments across sequential time points (Fig. 2a).

By applying a surgical cautery knife to one of the arterioles feeding into the corneal network, we were able to surgically induce a redistribution of blood flow throughout the neovessel network (Fig. 2a, light blue ‘x’). Blood flow redistribution was evidenced by the change in hemoglobin content in the neovessels (Fig. 2b, vessels with higher hemoglobin content (“*-C*↓*Hb*”) are depicted by lighter colors, according to the scale beneath each image). For example, one hour after intervention, corneal capillaries at the angiogenic front (below the “F”) demonstrated an increase in oxygenated hemoglobin, consistent with redistribution of blood flow through collateral arterioles in the network.

Over the first four days following cauterization, changes in blood flow occurred throughout the neovessel network (Fig. 2c). For example, in the vessel indicated in the inset (Fig. 2c, white arrow), the flow speed was diminished 48 hours after cauterization but returned to pre-intervention levels by 96 hours after cauterization. Over this time course, endothelial cell Tie2-GFP fluorescence intensity increased in some vessel segments (Fig. 2d, white arrow and vessel outlined in white in zoomed image below) following blood flow redistribution, but decreased in other segments (Fig. 2d, orange arrow and vessel outlined in orange in zoomed image below). A comparison of Tie2-GFP fluorescence intensity with WSS in individual neovessel segments (acquired by ICM and PAM, respectively), both prior to the surgically-induced blood flow redistribution (“Pre-intervention”, circles) and four days later (“Post-intervention”, squares) revealed a significant linear correlation between Tie2-GFP fluorescence intensity and WSS (R^2^ = 0.74), as confirmed by a parallel lines statistical test (Fig. 2e).

### Imaging of capillary hemodynamics and endothelial cell protein expression in individual neovessel segments before and after surgical redistribution of blood flow

After confirming that the linear correlation between Tie2-GFP fluorescence intensity and WSS in neovessel segments throughout the corneal network was preserved following surgical redistribution of blood flow, we sought to determine if the change in WSS in a given individual neovessel segment (resulting from the surgical redistribution of blood flow) was correlated with the change in Tie2-GFP fluorescence intensity in that same neovessel segment. Using the experimental protocol outlined in Fig. 2a and described above, we imaged the neovascular network of a stimulated (chemically burned) cornea in the Tie2-GFP mouse using BFM, ICM, and PAM seven days after applying the chemical burn. We then induced a surgical redistribution of blood flow (“intervention”) by cauterizing an arteriole feeding into the neovascular network. Two days after intervention, we re-imaged (using BFM, ICM, and PAM) the same field of view in order to visualize the same vessel segments.

**Figure 3:**
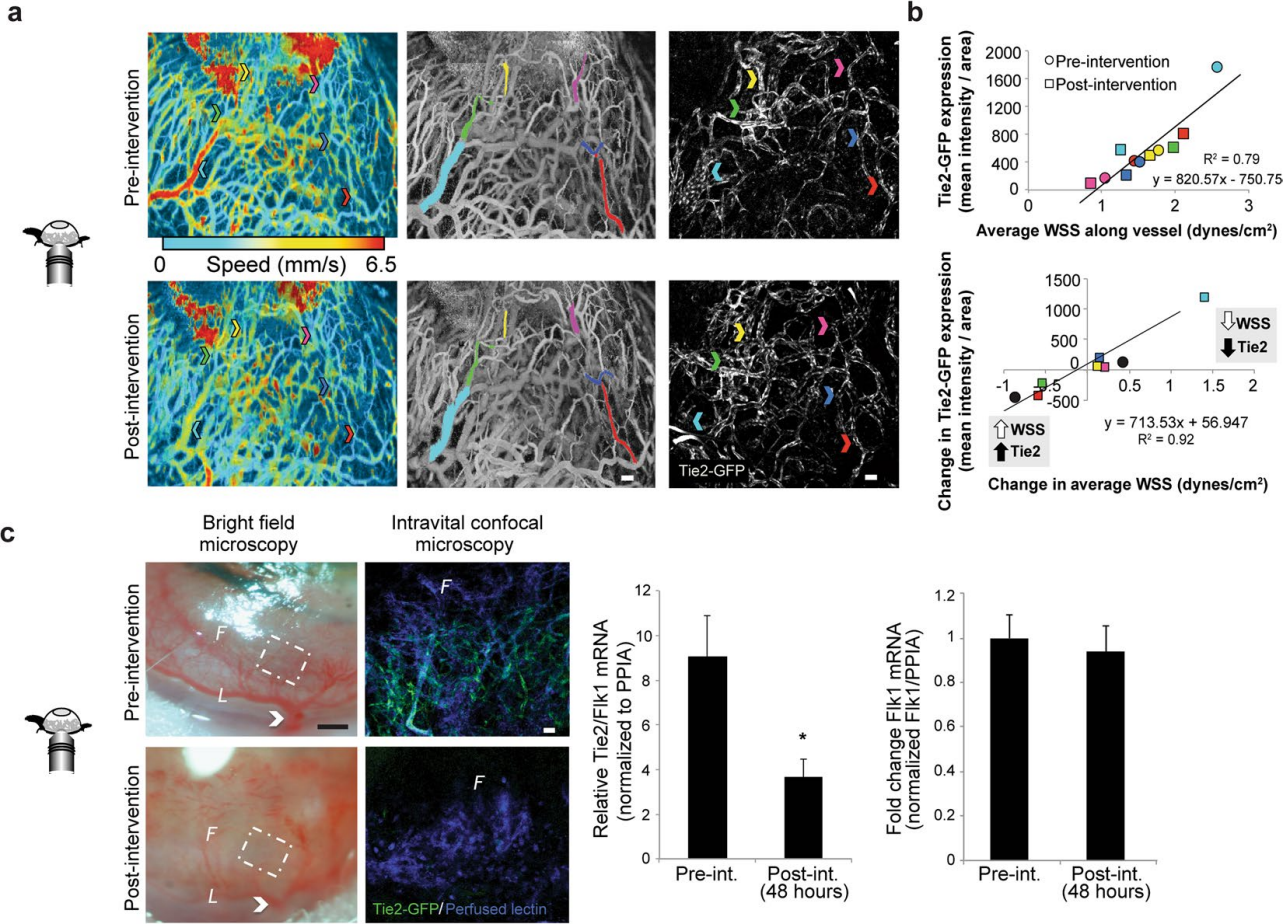
Representative example murine cornea analyzed using the dual imaging method demonstrates that endothelial cell Tie2-GFP fluorescence intensity was directly correlated with blood flow-induced WSS. (**a**) Changes in average WSS were correlated with changes in Tie2-GFP fluorescence intensity. Arrows indicate the color-matched vessel segments between PAM (center) and ICM (right). (**b**) The top graph shows the quantification of Tie2-GFP fluorescence intensity (mean intensity/pixel^2^) obtained using ICM versus blood flow-induced WSS (dynes per cm^2^) obtained using PAM. Measurements obtained pre-intervention are plotted as circles, measurements obtained post-intervention are plotted as squares, and each vessel segment that was analyzed is represented by the color that corresponds to the vessel segment highlighted in (**a**). The bottom graph reports the change in Tie2-GFP fluorescence intensity obtained using ICM versus the change in WSS obtained using PAM (both measurements are pre-ligated minus post-ligated values). (**c**) Bright field images of a representative cornea pre-intervention (top left) and 48 hours post-intervention (bottom left) show that blood flow is decreased throughout the network following an extensive surgical intervention. Right panel shows ICM images of the magnified vascular networks that are outlined by the white, dashed line boxes in the bright field images to the left, both pre- and 48 hours post-intervention. White arrow indicates the location of an arteriole branching off the limbus that was cauterized. Tie2 mRNA normalized to Flk1+ mRNA (left graph, n = 5 mice, p < 0.01) demonstrates a significant decrease in corneal Tie2 gene expression 48 hours after network-wide decrease in blood flow. In the same corneas, however, endothelial cell Flk1 mRNA (right graph, n = 5, p = 0.4) was unchanged. White scale bar indicates (**a**,**c**) 50 µm. Black scale bar indicates (**c**) 500 µm. 4.

As highlighted by colored arrows (first and third panels in Fig. 3a) and vessel outlines (second panel in Fig. 3a), the same individual vessel segments could be identified with both ICM and PAM before (“Pre-intervention”, top) and two days after (“Post-intervention”, bottom) surgical redistribution of blood flow (Fig. 3a, blood velocity is shown in first panel, with gray scale images of blood velocity shown in second panel). Tie2-GFP fluorescence intensity in individual neovessel segments within this corneal network correlated linearly with WSS, both before and two days after surgical redistribution of blood flow (Fig. 3b top, R^2^ = 0.79). Moreover, the change in blood flow-induced WSS in individual corneal neovessels after two days was directly proportional to the change in Tie2-GFP expression levels in those same neovessels (Fig. 3b bottom, R^2^ = 0.92). Specifically, when WSS in a given vessel segment decreased as a result of the surgical redistribution of blood flow, Tie2-GFP expression also decreased in that segment (e.g. blue square in Fig. 3b bottom). Conversely, when WSS in a vessel segment increased as a result of the surgical redistribution of blood flow, Tie2-GFP expression also increased in that segment (e.g. red square in Fig. 3b bottom). Data points in the upper-right quadrant indicate vessels that experienced a decrease in both WSS and Tie2-GFP fluorescence intensity. Data points in the bottom-left quadrant indicate vessels that experienced an increase in both WSS and Tie2-GFP fluorescence intensity.

Further confirmation of the correlation between Tie2-GFP expression and blood flow/WSS was obtained by more extensively reducing blood flow via cauterization of all visible feeder arterioles within the corneal angiogenic network. BFM images confirmed significantly diminished blood flow immediately following surgical intervention (Fig. 3c left). A network-wide decrease in Tie2-GFP fluorescence intensity was observed by ICM 48 hours later (p < 0.01, n = 5 corneas), and Tie2 mRNA levels in harvested corneas as measured by RT-PCR were also significantly diminished (p < 0.02, n = 5 corneas) at this time-point (Fig. 3c middle). The maintenance of constitutive Flk1 expression following this intervention implies that the reduction in Tie2-GFP was not due to loss of or general poor health of endothelial cells (Fig. 3c right). PPIA was used as housekeeping gene.

## Discussion

Heterogeneities in endothelial gene and protein expression have been observed at multiple levels of spatial scale throughout the blood circulation. These cell-to-cell^42^ and vessel-to-vessel^43^ differences have profound implications for the design of therapeutics that target the vasculature^44^ and for drugs that aim to ameliorate pathological vascular adaptations^45^. Although the topic of endothelial heterogeneity is an emerging focus of research^46^, the control systems that establish and maintain endothelial heterogeneities *in vivo*, and particularly in the microcirculation, have not yet been determined. Studies conducted in large vessels and in cultured monolayers of endothelial cells have demonstrated that endothelial cells are highly mechanosensitive, with fluid flow-induced shear stress serving as a regulator of endothelial cell gene expression^47^. Our novel observation of heterogeneous endothelial cell Tie2-GFP expression in murine corneal microvessels during angiogenesis motivated us to determine whether vessel-to-vessel differences in Tie2-GFP expression were dynamically coupled with differences in blood flow-induced shear stress. This required us to develop a new experimental platform for quantifying both vascular hemodynamics and endothelial cell protein expression in the same individual vessel segments throughout an *in vivo* microvascular network and across sequential time points. Employing dual-modality PAM and ICM imaging in an *in vivo* model of adult mammalian angiogenesis yielded two novel findings: 1) endothelial cell Tie2-GFP expression in an angiogenic network is linearly correlated with shear stress, and 2) local changes in shear stress are directly proportional to changes in endothelial cell Tie2-GFP expression in individual neovessel segments. To our knowledge, our study is the first to relate changes in endothelial cell protein expression within individual microvessel segments to changes in blood flow *in vivo*. Together, these findings implicate variable blood flow-induced shear stress levels throughout an angiogenic microvascular network as a dynamic driver of endothelial cell heterogeneity.

To our knowledge, our study is the first to use PAM to analyze functional hemodynamic changes in microvessels over time during adult corneal angiogenesis. The cornea permits detailed study of mechanisms of adult angiogenesis because it provides an avascular space that generates a reproducible angiogenic response^6, 48^ in which each neovessel segment is formed de novo and easily visualized using optical imaging approaches. The superficial location and largely planar growth of this angiogenic network makes it highly amenable to multi-modality imaging. In particular, the shallow depth of field allows high resolution imaging of dynamic changes in vessel structure at cellular resolution. In contrast, high resolution study of angiogenesis in other end-organ systems, such as the heart, can be particularly challenging given tissue depth and complex three-dimensional structure rendering them less amenable to ICM and PAM, which has a penetration depth of 1.2 mm^49^. Furthermore, PAM provides functional measurements of the microvasculature that supplement traditional metrics of vascular architecture, including blood flow velocity, hemoglobin content, and sO_2_. The use of PAM has allowed us to demonstrate that blood flow and hemoglobin content throughout the corneal angiogenic network is altered following limbal vessel cauterization. Vascular occlusions in the retina induce similar shunting of flow from the superior to inferior retinal circulation through pre-existing, but normally unperfused, collaterals^50, 51^. We believe this to be the first report of shunting of blood flow through collateral vessels within a corneal angiogenic network, a finding that should enable further study of the mechanisms that drive collateral recruitment. In addition to these acute flow changes, we also report for the first time secondary transitory flow changes seen over larger time courses, with alterations in flow occurring by 48 hours and taking up to 7 days to return to baseline. The prolonged timeline for these events should allow high resolution *in vivo* study of the compensatory response of endothelial cell and mural support cells to alterations in flow, also providing insight into how these accumulated changes can restore flow to baseline levels over time.

Prior studies of angiogenesis or vasculogenesis using intravital imaging have focused primarily on embryological development, predominantly using BFM and ICM. In the embryoid body model^52, 53^ and in the zebra fish^54^, for example, studies have elucidated events occurring a few hours or days after the initiation of blood flow^55, 56^. Our combined imaging technique should, in principle, be well suited for studying these early events because ICM and PAM are both compatible with the tissue thickness and required depth of field in these model systems. We further anticipate that the combination of ICM and PAM will be useful in studying microvascular adaptations that occur throughout adulthood and during the aging process, potentially providing time-lapse images of the same vessel or set of vessel segments within an expansive whole network over long time durations. While our study employed a GFP reporter mouse, additional applications could include fluorescently-tagged beads, therapeutic microspheres, DiI (1,1′-dioctadecyl-3,3,3′3′-tetramethylindocarbocyanine perchlorate) lipophilic membrane stain-labeled cells, and other lineage-tracing or reporter mice.

By demonstrating a dynamic and linear relationship between endothelial protein expression and blood flow-induced shear stress *in vivo*, our study serves as critical validation for published *in vitro* observations in cultured Human Umbilical Vascular Endothelial Cells (HUVECs) that link endothelial cell Tie2 expression to blood flow-induced WSS levels^20, 57–59^. Further, our findings are consistent with the notion that endothelial cells throughout the circulation exhibit a spectrum of maturation states^60, 61^ since endothelial cell Tie2 expression signifies phenotypic maturity^62^. Beyond validating previous *in vitro* observations, the data and imaging tools we present in this study may be leveraged by researchers who seek to build quantitative computational models of microvascular adaptations. Previous studies have demonstrated the dynamic, spatially-heterogeneous remodeling of collateral vessels after acute vessel ligation^63^. However, these data have not yet been coupled with genetic reporting, and our study provides unique and potentially valuable approach for parameterizing and validating computational models that are focused on evaluating mechanisms linking hemodynamics with gene expression in the microvasculature^22, 24–27^.

One caveat of our study is that we used relative GFP expression under the control of the Tie2-promoter as a surrogate for endothelial cell Tie2 expression. This assumption has been previously validated using an inducible GFP reporter in single cells where GFP fluorescence was shown to be directly proportional to GFP gene copy number and GFP mRNA expression. ICM can provide a sensitive and quantitative measure of endothelial cell protein expression over at least a 1,000 fold range^40^. However, it should be noted a lack of GFP signal may potentially reflect a fluorescence signal below the limits of ICM detection or a bias in photomultiplier threshold calibrated to higher expression levels, rather than a complete absence of Tie2 expression^40^. Furthermore, the strong positive correlation that we observed between endothelial cell Tie2-GFP and WSS in vessel segments does not prove that changes in WSS are sufficient to induce changes in endothelial cell Tie2-GFP expression. Indeed, there are likely a multitude of spatially and temporally altered regulators of Tie2 during angiogenesis, such as growth factors and the presence of hypoxia or inflammation in the tissue, that dynamically affect Tie2 expression^7, 64^. Therefore, future work is needed to determine whether changes in WSS cause changes in endothelial cell Tie2 expression, and if so, via what molecular mechanisms.

Tie2 has a known functional role in mural cell recruitment via Ang-1 signaling^65–67^. Therefore, one implication of our study is that changes in WSS during angiogenesis are correlated with the dynamics of mural cell recruitment through Tie2 expression and activation. However, it remains to be determined to what extent endothelial cell Tie2 expression levels relate to other structural and functional indicators of endothelial cell maturity, such as mural cell recruitment and vessel permeability alterations^7, 9, 66, 68, 69^, and to what extent those characteristics correlate with WSS levels. Moreover, studies performed on cultured endothelial cells have demonstrated that other signaling molecules implicated in angiogenesis and vessel maturation, including VEGF-R1, VEGF-R2, and VE-Cadherin, are also affected by WSS levels^20, 58^. Therefore, it would be interesting to apply the combination of PAM and ICM to other *in vivo* model systems where it is possible to visualize endothelial cell expression of these (and other) relevant genes and proteins. Nonetheless, the study of Tie2-Ang signaling in endothelial cells has inspired both pre-clinical and clinical studies^28–30^ designed to evaluate a drug, VE-PTP, which increases phosphorylation of Tie2 even in the presence of high Angiopoietin-2 (Ang-2) levels. However, the impact of this drug on the endothelium’s ability to respond to signals associated with injury and disease, including blood flow shunting or hemodynamic alterations during angiogenesis, has not yet been examined. Indeed, no study to date has attempted to ascertain the efficacy of VE-PTP given the endothelial heterogeneity that we have demonstrated with respect to Tie2 expression and its relationship to biomechanical cues. Our experimental platform would enable this and other similarly informative studies to be completed in *in vivo* disease models.

In conclusion, we have presented a new experimental platform that uniquely combines PAM and ICM to obtain quantitative, high-resolution information about both hemodynamic parameters and protein expression levels in individual capillary segments during adult mammalian angiogenesis. Understanding how biomechanical and biochemical stimuli integrate with one another to drive the dynamic, multi-cell process of angiogenesis and vessel maturation is key to developing effective therapies for controlling these processes, which are essential to tissue function, healing, and regeneration. We envision that this approach will be most useful for placing cell-level changes in the context of local hemodynamic alterations, which will facilitate discoveries of how spatial and temporal phenotypic heterogeneities arise and persist *in vivo* during vascular remodeling and disease.

## Methods

### Experimental Animals

All surgical procedures were approved by the Institutional Animal Care and Use Committee at the University of Virginia, and completed in accordance with our approved protocol under these guidelines and regulations. We used transgenic Tie2-GFP mice from The Jackson Laboratory (Stock Tg(TIE2GFP)287Sato/J, Stock Number 003658, Bar Harbor, ME). The number of mice per figure is reported in each figure caption. All mice were 8–24 weeks of age at time of manipulation, with treatment and control groups age-matched within each experiment.

### Cornea Alkali Burn

The corneal alkali burn model was adapted from Suvarnamani C *et al*.^6, 70^ Briefly, animals were anesthetized with an intraperitoneal injection of ketamine/xylazine/atropine (60/4/0.2 mg/kg body weight). A drop of sterile 0.5% Proparacaine Hydrochloride Ophthalmic Solution (Henry Schein Inc; Melville, NY) was added as a topical anesthetic to numb the eye before treatment. The right eye was chemically cauterized by pressing applicator sticks coated with 75% AgNO_3_/25% KNO_3_ to the cornea (SnyptStix by Grafco; Atlanta, GA) for five seconds while the animals were anesthetized. Excess silver nitrate was removed by rinsing corneas with 0.9% NaCl saline solution (Healthmark Services, Montreal, Quebec, Canada). An additional drop of Proparacaine was applied to each cornea post-treatment as a topical anesthesia. All treatments were conducted while the mouse was on a heat source to maintain constant body temperature. At the terminal end point, mice were humanely euthanized by carbon dioxide asphyxiation.

### Blood Flow Intervention

Blood flow was dynamically altered throughout the corneal neovascular networks by cauterizing of a primary feeder arteriole, thus preventing flow through this vessel. Cauterization was accomplished using a Bovie Fine Tip Cautery knife (Aaron Medical, Clearwater, FL), with one or more of the multiple small, feeder arterioles being sealed with diathermy.

### Intravital Cornea Imaging

Intravital corneal microscopy was accomplished through modification of the technique described by Di Girolamo *et al*.^71^ Briefly, animals were anesthetized with an intraperitoneal injection of ketamine/xylazine/atropine (60/4/0.2 mg/kg body weight)(Zoetis; Kalamazoo, MI/West-Ward; Eatontown, NJ/Lloyd Laboratories; Shenandoah, IA). A drop of sterile 0.5% Proparacaine Hydrochloride Ophthalmic Solution was added as a topical anesthetic to numb the eye before imaging. To allow visualization of vascular endothelium, anesthetized mice were administered a retro-orbital injection of labeled isolectin^72^ (IB4-Alexa647; Life Technologies, Carlsbad, CA) 10 minutes before imaging began. Digital images of the corneal neovasculature were acquired using confocal microscopy (Nikon Instruments Incorporated, Melville, NY; Model TE200-E2; 10X, 20X,and 60X objectives). Three to four fields of view per cornea were imaged at each time point, and full-thickness Z-stack (2–5 μm step cells) volume renders were used to capture the entire thickness of the corneal neovascular network in each field of view. Genteal gel was applied to the eye during imaging to prevent drying. Mice were placed on a microscope stage that contained a warming pad to maintain a constant body temperature of 37°C, eyelashes and whiskers were gently pushed back with ophthalmic lubricant Genteal gel (Alcon; Forth Worth, TX), and the snout was gently restrained with a nosecone. During imaging, the eye was placed against a coverslip that rested on the stage of the inverted confocal microscope.

Corneas were imaged prior to the burn injury, 2 days after the burn injury, and 7 days after the burn injury. In the subset of mice that received a subsequent cautery-induced blood flow redistribution, fine needle tip diathermy was performed 7 days after initial burn injury and corneas were imaged 48 hours and also 4 days following cauterization. Validation of GFP signal and separation from background autofluorescence was determined by imaging both uninjured contralateral cornea, as well as imaging of corneal burns performed in C57Bl6 WT non-GFP expressing mice (not shown).

### Bright Field Imaging

Bright field images of corneas under 4X magnification were obtained using a Nikon Digital Sight DS-L2 Camera Controller (Nikon Instruments Inc, Melville, NY; Model 214602) to assess the network-wide hierarchy of neovessels and determine the presence or absence of blood flow in individual vessel segments. For each mouse, multiple fields of view were taken encompassing the entire circumference of the eye.

### Photoacoustic Microscopy

Optical-resolution, multi-parametric photoacoustic microscopic (PAM) images of the angiogenic corneal networks were acquired as thoroughly described in our previous literature^73, 74^. This technique enabled simultaneous quantification of microvascular diameter, oxygen saturation of hemoglobin (sO_2_), and blood flow within individual vessel segments^73^. In particular, a temporal decorrelation approach has been developed and optimized for quantifying blood flow by both our group and others^73, 75^. Prior work establishing PAM has demonstrated that we can correctly measures blood flow speeds within the range of 0.18–21 mm/s^73^, which well covers the reported murine physiologic capillary flow velocity of 0.75–6.75 mm/s^76^.

Consistent with prior work, in this study we acquired images using two laser wavelengths, 532 nm and 559 nm. The applied laser pulse energies at the corneal surface were 50 nJ and 30 nJ at 532 nm and 559 nm, respectively. The laser beams were through a doublet onto the cornea with a beam diameter of 2.7 µm. Two-dimensional raster scans were performed over the regions of interest; scans of the entire cornea took approximately 30 minutes. It is worth noting that the temporal decorrelation method is primarily sensitive to the blood flow in capillaries that are perpendicular to the optical axis (i.e. transverse blood flow). Fortunately, the corneal vascular network is a very planar structure that has few penetrating capillaries. Therefore, the approximate angle between the measured capillaries and the optical axis of the PAM system was 90 degrees. To ensure the correct orientation of the vessel bed, the network was carefully pre-imaged and aligned to be perpendicular to the optical axis before we conducted obtained measurements for quantification. Throughout the experiment, the mouse was maintained under anesthesia with 2.0% vaporized isoflurane and the body temperature was kept at 37 °C.

After obtaining the measurements using a single scan, our system could provide the same spatial resolution for all the measurements, which is ~2.7 μm, as tested in our previous study outlining the PAM system^73^. After we acquired the measured value in each pixels, a 10 μm × 10 μm averaging window, which is close to the average diameter of the capillaries, was used to suppress the fluctuation in the measurements. No additional image processing or repeated measurements were utilized.

For corneas undergoing burn injury, PAM and ICM was performed 2 days and 5–7 days after burn injury. For corneas that underwent burn injury and subsequent vessel cauterization, PAM and ICM were performed prior to the cautery-induced blood flow redistribution, one hour after intervention, and 48 hours after intervention.

### RT-PCR

In order to determine if regulation of Tie2 is observed at the gene level, we enucleated the eyes to harvest the cornea for RT-PCR analysis (n = 5 mice). We harvested corneas for analysis prior to cautery-induced blood flow redistribution and two days after performing cautery-induced blood flow redistribution. In an attempt to maximize the extent of blood flow redistribution, we cauterized every major arteriole feeding the corneal neovascular network. RNA extraction was conducted with the PureLink RNA Micro Scale Kit, followed by CDNA generation with a SuperScript cDNA Synthesis Kit and RT-PCR using SensiMix II Probe (Bioline; Taunton, MA). PCR was run on a Bio-Rad CFX96 Detection System. We measured Tie2 gene expression [Primer 1–5′-GCCTCCTAAGCTAACAATCTCC-3′; Primer 2–5′-GATGGCAATCGAATCACTGAAC-3′] normalized to constitutively-expressed Flk1 [Primer 1–5′-GGATCTTGAGTTCAGACATGAGG-3′; Primer 2–5′-GGAATTGACAAGACAGCGACT-3′] to control to the number of endothelial cells within the tissue and also assess the general health of the network in the event that cauterization induced excessive endothelial cell dysfunction^20^. We also utilized the housekeeping gene, PPIA [Primer 1–5′-TTCACCTTCCCAAAGACCAC-3′; Primer 2–5′-CAAACACAAACGGTTCCCAG-3′] as control^77^ (all primers and probe were purchased from Integrated DNA Technologies, Coralville, IA) Calculations for comparing the expression levels of two genes from the same cell samples were performed as previously described^78, 79^.

### Quantification of Microvascular Outgrowth, Tie2-GFP Expression, and Shear Stress

ImageJ (NIH, Bethesda, MD) imaging software was used to quantify vessel lengths, vessel segment area, vascular network areas, and mean pixel intensities in both confocal and bright field images. PT Gui (New House Internet Services BV; Rotterdam, The Netherlands) software was used to overlay the ICM image onto PAM image, providing good alignment between Tie2 fluorescence and corresponding flow velocity maps in the same vessel segment through use of manually specified control points. When analyzing vessel segments imaged via ICM and PAM, we analyzed at least two fields of view with at least ten candidate vessels per cornea at 2 days after burn injury, 7 days after burn injury, 2 days after cautery-induced blood flow redistribution, and 7 days after cautery-induced blood flow redistribution.

GFP was imaged using an excitation wavelength of 488 nm, and perfused lectin was imaged using an excitation wavelength of 647 nm. Gain settings on the confocal microscope were held constant throughout the experiment. Vessel segments were outlined using the draw tool in ImageJ, and both the mean and raw GFP fluorescence intensity were measured, as well as the vessel area and vessel length. Background auto fluorescence of the cornea was determined by measuring and averaging fluorescence intensity in three regions absent of vessels. Then, this value was subtracted from the fluorescence intensity density for each vessel segment.

Neocapillary segments chosen for analysis were in focus in both imaging modalities, aligned with the PAM image plane (i.e., ran perpendicular to the optical axis), exhibited blood flow in PAM and lectin perfusion in ICM, and were fully contained in regions of interest for both imaging modalities. Ten vessel segments per cornea, sampled from different locations throughout the corneal neovascular networks, from 4 different mice were identified in both PAM and ICM images and analyzed using a custom analysis algorithm run within Matlab based on previously established algorithms to calculate WSS^80^. Briefly WSS within each vessel segment was calculated according to the measured diameter and flow from PAM using the general formula

1 $$shear\,stress=8\cdot \mu \cdot \frac{v}{d}$$

where *v* indicates the blood velocity, *d* indicates the vessel diameter, and *μ* indicates the blood viscosity (assumed to be 10 cP)^80^.

Average GFP expression intensity density over the vessel segment versus WSS in the same vessel segment was plotted for each analyzed vessel segment. Plots indicate at which time point the data were collected. In networks where measurements of blood flow were obtained pre- and post intervention, the change in Tie2-GFP expression was plotted against the change in WSS, and the R^2^ value was reported.

### Statistical Analysis

Correlation between WSS and Tie2 gene expression was calculated by a goodness-of-fit linear regression and reported as the co-efficient of determination (R^2^). RT-PCR data was compared using Student’s t-test. Significance was asserted at p < 0.05, and data are presented as mean +/− standard error.

### Data Availability

All data generated or analysed during this study are included in this published article. Any inquiries are available from the corresponding author on reasonable request.
